# Complex Intratendinous Pectoralis Major Rupture After Heavy Bench Press

**DOI:** 10.7759/cureus.112354

**Published:** 2026-07-09

**Authors:** Ryan M Magee, Laurel A Marsh, Gabriella D Fields, Jason W Deck

**Affiliations:** 1 Department of Family and Sports Medicine, University of Oklahoma School of Community Medicine, Tulsa, USA

**Keywords:** bench press injury, cortical button fixation, intratendinous tear, magnetic resonance imaging, myotendinous junction, pectoralis major rupture, sports medicine, tendon injury, weightlifting injury

## Abstract

A pectoralis major rupture is a rare injury with important strength and cosmetic consequences, most often described in young, active men after a high-load eccentric contraction during the heavy bench press. Magnetic resonance imaging (MRI) is commonly used to confirm the diagnosis and localize the tear, but the location described on MRI may not fully define tendon continuity, tissue quality, or the tendon available for fixation. We report the case of a 23-year-old male powerlifter who developed acute right chest pain, swelling, ecchymosis, deformity, and pectoralis retraction during a 500-lb bench press one-repetition maximum attempt. Preoperative MRI showed a high-grade injury of the sternal head fibers of the right pectoralis major and was interpreted as a grade 3 myotendinous tear with a distal tendon stump measuring approximately 30 mm. The absence of an edema-like signal around the stump supported, but did not prove, a more proximal myotendinous injury with a preserved distal tendon.

During surgery, however, the repairable tendon attached to the retracted muscle was more limited than the MRI impression suggested. Exploration showed intratendinous disruption, with only a small tendon remnant attached to the retracted muscle and substantial tendon remaining at the humeral footprint. This changed the repair problem from simple fixation of a robust distal stump to mobilization of limited tendon and adjacent myotendinous tissue, locking whipstitch fixation with high-strength suture tape, unicortical humeral drilling, cortical button fixation, and restoration of the axillary fold and pectoralis contour. At approximately three months, the patient had a full range of motion, near-normal pectoralis strength, preserved contour, and no documented pain, infection, neurovascular deficit, wound dehiscence, rerupture, or other major early complication. This short follow-up cannot establish long-term repair integrity or safe return to maximal bench press. In this patient, MRI identified the high-grade rupture, while intraoperative inspection clarified the repairable tendon available on the retracted muscle.

## Introduction

Pectoralis major rupture is rare, but reported cases have increased with participation in weight training and strength sports. The typical mechanism is a high-load eccentric contraction of the pectoralis major, often with the shoulder abducted and externally rotated, making the heavy bench press a classic setting for injury. Reported rupture patterns vary by chronicity, involved muscle segment, tear extent, and anatomic location; locations include injury at the muscle belly, myotendinous junction, intratendinous region, and humeral insertion [1-3]. These distinctions affect operative management because tear location, tendon continuity, tendon quality, and tissue mobility influence exposure, mobilization, fixation choice, and rehabilitation.

Magnetic resonance imaging (MRI) is commonly used to confirm pectoralis major rupture, define the involved muscle segments, assess retraction, and guide treatment. However, MRI findings such as edema, retraction, and an apparent distal tendon stump may not fully define tendon continuity or whether enough tendon remains on the retracted muscle for standard tendon-to-bone fixation. Prior studies have described inconsistent classification of pectoralis major tears, imaging pitfalls, and imperfect correlation among clinical assessment, imaging findings, and treatment strategy [1,3-5]. This case describes a high-load pectoralis major rupture in which MRI suggested a primarily myotendinous sternal head tear, whereas surgery revealed intratendinous disruption with limited repairable tendon attached to the retracted muscle. The main contribution is not that MRI missed the rupture, but that classification and repair planning were affected by the tendon actually available at surgery.

## Case presentation

A 23-year-old male powerlifter with no relevant medical or surgical history presented to orthopedic urgent care four days after an acute right chest injury during a 500-lb bench press one-repetition maximum attempt. During the lift, he felt a pulling and popping sensation across the right chest, followed by pain, swelling, bruising, deformity, and visible retraction of the right pectoralis contour. He reported weakness and discomfort with cross-body adduction of the right upper extremity, with less discomfort during internal rotation. He had partial pain relief with over-the-counter ibuprofen. He denied numbness or tingling.

Initial examination showed swelling and tenderness of the right chest wall, diffuse ecchymosis extending from the right lateral chest wall to the right upper arm, visible pectoralis major retraction, and a soft-tissue mass palpable just medial to the axilla. Strength was decreased with cross-body adduction and internal rotation compared with the contralateral side. Sensation was intact, and no open wound was present. MRI was ordered, supportive sling use was discussed, and sports medicine follow-up was arranged.

At sports medicine evaluation one week after injury, examination showed ecchymosis and swelling extending into the axilla, inferior displacement of the nipple on the injured side, a retracted pectoralis major tendon palpable just medial to the axillary fold, thinning of the anterior axillary fold, 4+ internal rotation strength on manual testing, and intact neurovascular status. This 4+ internal rotation strength reflected the sports medicine examination finding and was not used as a claim of improvement from the urgent care visit. Right shoulder four-view radiographs showed type II acromial morphology and were otherwise negative.

An MRI of the chest without contrast was performed six days after injury. Evaluation was moderately limited by breathing motion. Osseous structures showed normal magnetic resonance signals without marrow edema. The report described signal abnormality, irregularity, and retraction along the lateral margin of the sternal head portion of the pectoralis major. A mild edema-like signal was present in the clavicular head fibers, although most clavicular head fibers were intact. The sternal head tear was interpreted as primarily myotendinous, with a distal tendon stump measuring 30 mm. No edema-like signal was present around this tendon stump, which supported a more proximal myotendinous tear. There was no significant anterior subluxation of the biceps tendon, also supporting a myotendinous injury, although involvement of the more distal posterior laminar fibers was considered possible. Using the ElMaraghy and Devereaux framework, the injury was discussed in terms of acute timing, sternal-head involvement, tear extent, and location along the myotendinous-to-tendinous region [1]. The radiologic impression was a grade 3 myotendinous tear of the sternal head fibers of the right pectoralis major, with a small area of grades 1-2 strain involving the clavicular head fibers. Because motion limited the study, the MRI was interpreted cautiously for tendon continuity and for the amount of tendon remaining attached to the retracted muscle. Representative axial and coronal magnetic resonance images are shown in Figures 1-2.

**Figure 1:**
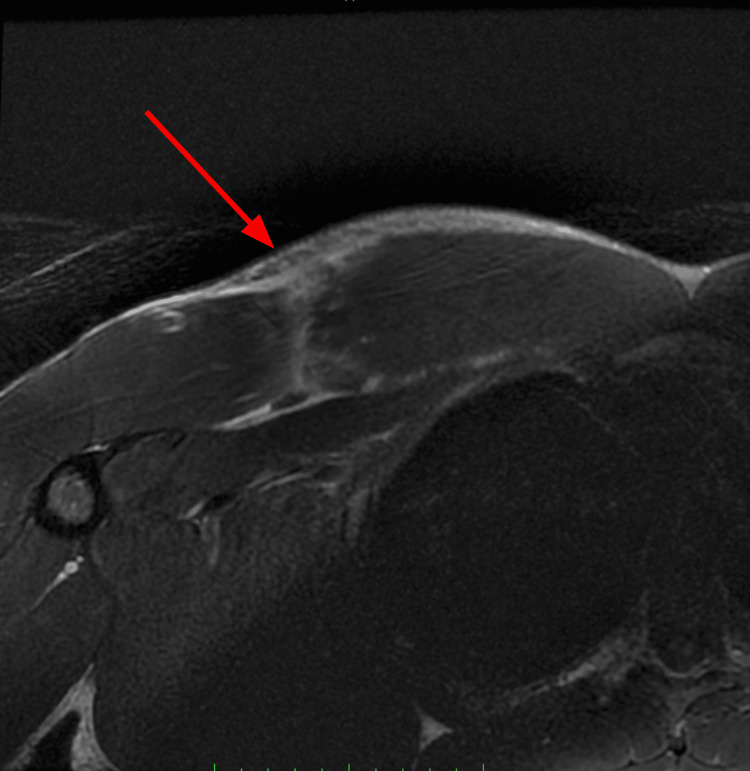
Axial magnetic resonance image of the right pectoralis major injury. Axial magnetic resonance image of the right pectoralis major region demonstrating abnormal signal, contour disruption, and retraction along the lateral sternal-head fibers near the anterior axillary fold highlighted by the arrow. The humeral-sided insertional region was interpreted in conjunction with the full MRI series and clinical examination. The preoperative interpretation favored a predominantly myotendinous sternal-head injury, although motion limitation reduced certainty about tendon continuity. Operative exploration later showed intratendinous disruption with limited repairable tendon attached to the retracted muscle.

**Figure 2:**
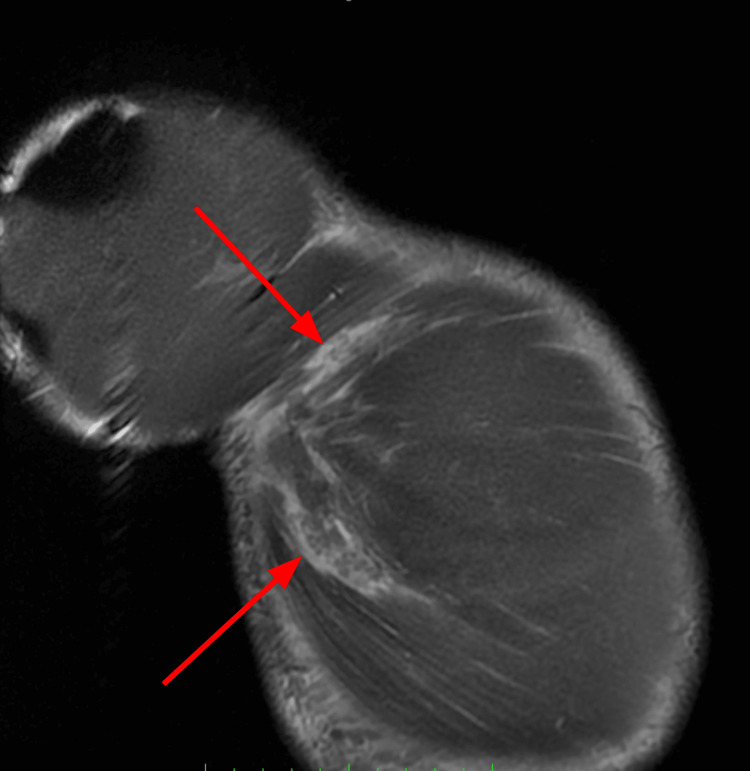
Coronal magnetic resonance image of the right pectoralis major injury. Coronal magnetic resonance image demonstrating edema-like signal and retraction involving the right pectoralis major sternal-head region, highlighted by the arrows. The radiologic impression favored a grade 3 myotendinous tear with a distal tendon stump measuring approximately 30 mm and mild grades 1-2 strains of the clavicular head fibers. Because the study was motion-limited, the MRI was interpreted cautiously for tendon continuity and the amount of tendon remaining on the retracted muscle. Intraoperative findings showed intratendinous disruption requiring extensive mobilization, locking whipstitch fixation through limited tendon and adjacent myotendinous tissue, and cortical button fixation.

After discussion of operative and nonoperative treatment options, operative technique, recovery, rehabilitation, and surgical timing, the patient elected surgical repair. He underwent right open pectoralis major repair 12 days after the injury. Intraoperatively, the tear was more complex than a simple myotendinous tear with a robust distal stump available for repair. Exploration showed a complex intratendinous rupture, with only a small tendon remnant attached to the retracted muscle and substantial tendon remaining at the humeral footprint. The humeral-sided tendon material was preserved because complete mobilization of that tissue risked further weakening the limited tendon remaining on the muscle side. The remaining tendon and adjacent myotendinous tissue were extensively whipstitched using locking loops of FiberTape (Arthrex, Inc., Naples, Florida).

The humeral footprint of the pectoralis major tendon was identified, and two unicortical drill holes were placed along the lateral margin of the footprint. Fluoroscopy confirmed appropriate tunnel position. The sternal head sutures and tendon were passed deep to the clavicular head and brought to the lateral window of the deltopectoral interval. Pectoralis repair buttons (Arthrex, Inc., Naples, Florida) were deployed through the unicortical tunnels. The sutures were tensioned toward the prepared footprint and secured. The repair restored the axillary fold and pectoralis contour and brought the tendon back toward the humeral footprint. The intratendinous tear pattern and retraction required more extensive mobilization than would be expected for a straightforward humeral-sided avulsion or a robust distal tendon stump.

At eight days postoperatively, the patient was doing well with no pain. Examination showed a clean, dry, and intact incision; good axillary contour; good pectoralis major contour; intact neurovascular status; and a small seroma on the lateral border of the axillary fold. Right shoulder three-view radiographs showed postsurgical changes consistent with the repair, with appropriate unicortical button and tunnel placement. The patient remained nonweightbearing in a sling, with a home exercise program and gentle range of motion exercises planned for six weeks.

At the six-week follow-up, the patient was doing well with no pain or complaints and had not yet started physical therapy. Examination showed a healing incision, 150 degrees of forward elevation, 105 degrees of abduction, 45 degrees of external rotation, good axillary contour, good pectoralis major contour, and intact neurovascular status. Weight-bearing was advanced, the sling was discontinued, and formal physical therapy using a pectoralis major repair protocol was initiated.

At approximately three-month follow-up, the patient continued to report no pain and no complaints. He had been working with a physical therapist on gradual return to gym activity. Examination showed a healing incision, full range of motion, near-normal pectoralis strength, good axillary contour, good pectoralis major contour, and intact neurovascular status. He was cleared to advance gym activities and slowly advance weight, with a planned follow-up in three months for likely final reassessment. No infection, neurovascular deficit, wound dehiscence, rerupture, or other major complication was documented during early follow-up.

## Discussion

The principal finding in this case is that MRI correctly identified a high-grade pectoralis major rupture, but intraoperative inspection changed the repair problem. Preoperative MRI suggested a primarily myotendinous sternal-head tear with an apparent distal tendon stump. Surgery showed that the tendon attached to the retracted muscle was limited, while substantial tendon remained at the humeral footprint. This distinction directly affected fixation because the repair required mobilization and suture fixation through limited tendon and adjacent myotendinous tissue rather than simple repair of a robust distal stump.

The MRI interpretation was reasonable based on the reported findings. The absence of edema-like signal around the distal tendon stump and the lack of significant anterior biceps tendon subluxation supported a more proximal myotendinous injury. The report also noted that the examination was moderately limited by breathing motion and that involvement of the more distal posterior laminar fibers was possible. These findings do not mean the MRI failed to diagnose the rupture. Rather, MRI diagnosed the injury, while surgical exploration clarified tendon continuity, tissue quality, and the tendon available for repair.

Tear-location terms are useful, but location alone may not describe the repair problem. The ElMaraghy and Devereaux classification framework incorporates injury timing, location, tear extent, and standardized terminology, and later work has also attempted to refine classification for sternocostal-head tears [1,6]. However, classification systems are inconsistently applied, and even accurate location terminology may not fully convey tendon length, tendon quality, mobility, or the ability of the remaining tissue to hold sutures. In addition to tear location, reports should describe whether a usable tendon remnant was present on the retracted muscle and whether the tear behaved like a standard tendon-to-bone repair or a more complex intratendinous/junctional injury.

Repair strategy depends on the tissue available at surgery. In a typical humeral-sided avulsion, the operative goal is usually anatomic reattachment of the tendon to the humeral footprint. In an intratendinous or junctional tear, the surgeon may instead face limited tendon length, poor tendon substance, scarred tissue, or a weaker suture-tendon interface. Biomechanical studies have shown that repair constructs are weaker than the native tendon attachment and that failure may occur through the tendon-suture interface [7,8]. In this case, locking whipstitch fixation with high-strength suture tape and cortical buttons was used to distribute load through the remaining tendon and adjacent myotendinous tissue while restoring the contour of the pectoralis major.

Available outcome data generally support operative repair for many young active patients with complete pectoralis major rupture, particularly when strength, cosmesis, and return to activity are priorities [9,10]. However, many available studies primarily compare operative timing, fixation method, or operative versus nonoperative treatment, and they variably report tear location and tissue quality. Reports of irreparable junctional or intrasubstance pectoralis major tears suggest that medial or intratendinous tear patterns can create reconstructive problems that differ from standard insertional avulsions [11]. The literature describes imaging pitfalls and imperfect correlation among clinical assessment, imaging findings, and treatment strategy, but precise estimates of how often MRI and operative findings differ specifically in tendon continuity or repairable tendon availability remain limited [3-5].

The practical lesson is not that MRI should be considered unreliable. MRI remains essential for confirming the diagnosis, defining the involved muscle segments, evaluating retraction, and guiding operative planning. The lesson is narrower: when MRI shows a high-grade tear with significant retraction, motion limitation, possible posterior laminar involvement, or uncertainty about tendon continuity, surgeons should be prepared for a more complex repair than the apparent tear location alone suggests. Surgical planning should account for the possibility of limited repairable tendons, the need for extensive mobilization, and a modified fixation strategy.

This case report has limitations. It describes a single patient and cannot estimate how often MRI and operative findings differ in pectoralis major rupture. Intraoperative photographs were not available for publication, limiting visual demonstration of the lesion and surgical technique. Follow-up is limited to approximately three months, and no formal isokinetic strength testing, validated patient-reported outcome score, or long-term return-to-maximal-lifting outcome was documented. Early recovery was favorable, with no documented pain, full range of motion, preserved axillary and pectoralis contour, and near-normal pectoralis strength at approximately three months. However, this short follow-up cannot establish long-term repair integrity, safe return to heavy bench pressing, or risk of rerupture. Longer follow-up is needed to describe durability of repair and return to maximal or near-maximal lifting.

## Conclusions

MRI is essential for confirming pectoralis major rupture and guiding operative planning, but it may not fully define tendon continuity, tendon quality, or the amount of repairable tendon attached to the retracted muscle. In this case, MRI suggested a primarily myotendinous sternal-head tear with a preserved distal tendon stump, while surgery showed intratendinous disruption with only a small tendon remnant attached to the retracted muscle and substantial tendon remaining at the humeral footprint. When imaging shows significant retraction or complex tear morphology, surgeons should be prepared for limited repairable tendon and a modified fixation strategy. Future reports should describe not only tear location but also tendon continuity, tendon quality, and whether enough tendon is present for standard tendon-to-bone fixation.
